# New-Onset Guttate Psoriasis Induced by Durvalumab: Potential Role of Bisoprolol

**DOI:** 10.7759/cureus.97733

**Published:** 2025-11-25

**Authors:** Olivia Tshika Wa Kabeya, Dieu-Donné Kaziga Wiyaou, Ibtissam Moustaghfir

**Affiliations:** 1 Department of Dermatology, Cliniques Universitaires de Kinshasa, Kinshasa, COD; 2 Department of Dermatology, Centre Hospitalier de Montluçon, Montluçon, FRA; 3 Department of Cardiology, Centre Hospitalier de Montluçon, Montluçon, FRA

**Keywords:** anti-pd-l1, bisoprolol, cutaneous toxicity, durvalumab, guttate psoriasis

## Abstract

Durvalumab is an immune checkpoint inhibitor targeting programmed death-ligand 1 (PD-L1), used in the treatment of various advanced cancers. Immune checkpoint inhibitors are associated with immune-related adverse events, mainly affecting the skin and digestive system. Immune-mediated psoriasis has been observed in patients treated with durvalumab. We report a case of new-onset psoriasis in a patient treated concomitantly with durvalumab and bisoprolol, prescribed for a well-differentiated keratinizing squamous cell carcinoma of the upper right lung and for sinus tachycardia, respectively. Topical therapy was effective, and both durvalumab and bisoprolol were continued. The potential role of bisoprolol, a beta-blocker known to induce or exacerbate psoriasis, is discussed. This case highlights the importance of identifying potential drug interactions that may trigger psoriasis.

## Introduction

Immune checkpoint inhibitors (ICIs) have revolutionized the management and prognosis of patients with various cancers. Among them, durvalumab, an anti-programmed death-ligand 1 (PD-L1) monoclonal antibody, has shown efficacy in the treatment of lung cancer [1,2]. Although ICIs offer a more favorable safety profile compared to chemotherapy, they may cause immune-mediated adverse events through CD4+/CD8+ T-cell activation and proliferation [3]. These toxicities can affect multiple organ systems, with cutaneous manifestations being the most frequent and often appearing early during treatment [4].

More than one-third of treated patients may develop maculopapular exanthema, pruritus, lichenoid eruptions, or vitiligo [5]. psoriasiform eruption is a rare immune-related adverse event. It usually represents a flare of pre-existing disease, but de novo psoriasis may also occur, typically after several months of therapy [5].

Bisoprolol, a cardioselective beta-blocker acting on cardiac β1-adrenergic receptors, is commonly prescribed for arterial hypertension, arrhythmias, heart failure, and angina. It has been associated with cases of drug-induced psoriasis, likely through disruption of intracellular signaling pathways in keratinocytes [6].

New-onset psoriasis induced by durvalumab has been reported [7,8]. Similarly, psoriasis has also been described with bisoprolol [9]. However, to our knowledge, no cases of psoriasis induced by durvalumab in a patient receiving bisoprolol have been reported. We therefore present a case of new-onset guttate psoriasis in a patient treated with durvalumab and bisoprolol.

## Case presentation

**Figure 1:**
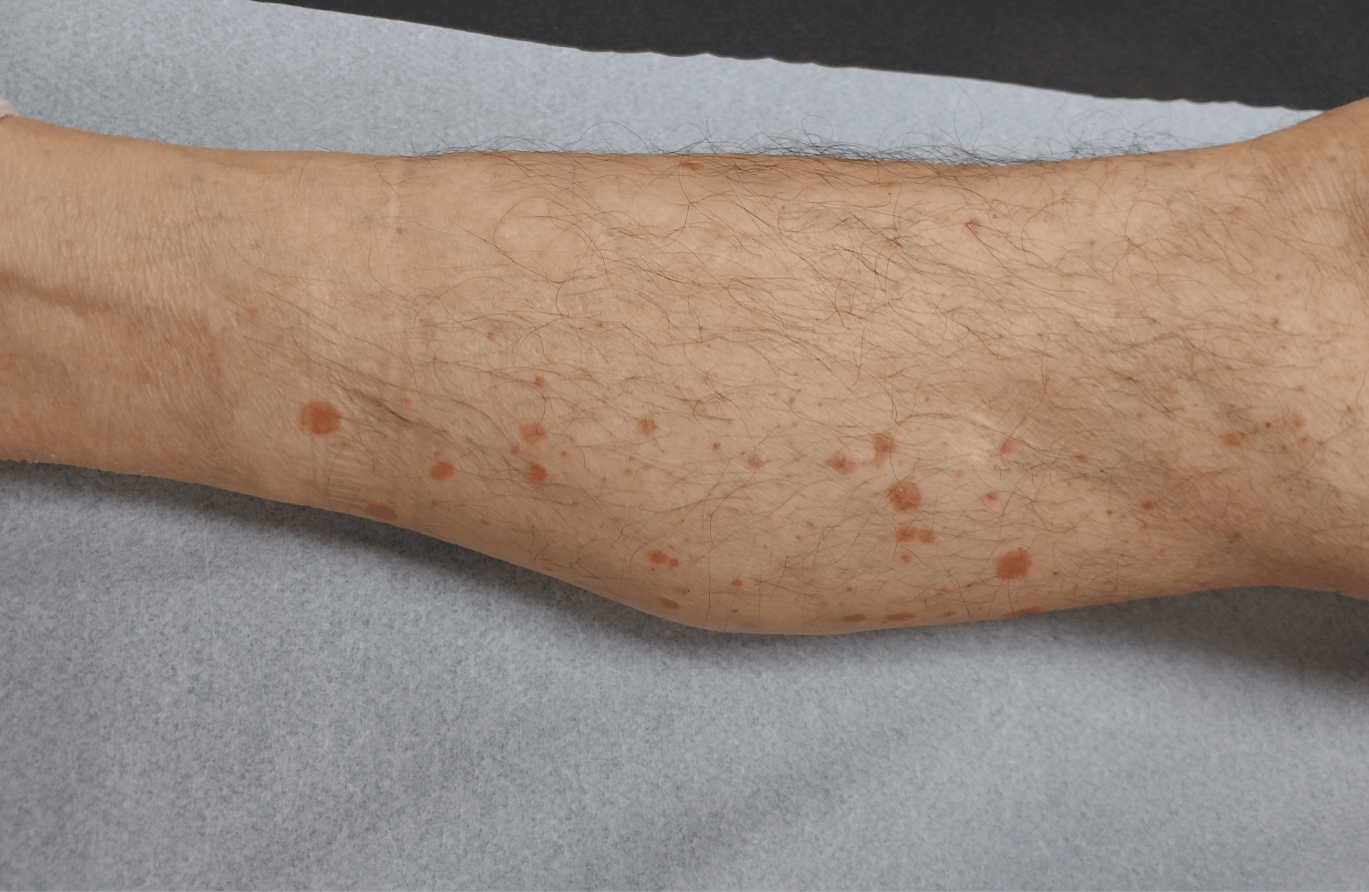
Localized erythematosquamous plaques on the right leg

We report the case of a 73-year-old patient under oncologic follow-up for a well-differentiated keratinizing squamous cell carcinoma of the right upper lung lobe, stage III, treated with durvalumab as second-line therapy following chemoradiation. Three months prior to initiating immunotherapy, a sinus tachycardia prompted the introduction of bisoprolol at a dose of 2.5 mg twice daily. The patient had no personal or family history of psoriasis, nor any recent infection, vaccination, or new medication. Ten days after the first infusion of durvalumab, the patient was referred for dermatologic evaluation due to the onset of a cutaneous eruption. Clinical examination revealed small, well-demarcated, mildly thickened erythematosquamous plaques, symmetrically distributed over the legs, elbows, back, scalp, and face. Skin involvement was estimated at 20% of the body surface area (BSA), with a Psoriasis Area and Severity Index (PASI) score of 4 and an Investigator’s Global Assessment (IGA) score of 2 (Figure 1).

**Figure 2:**
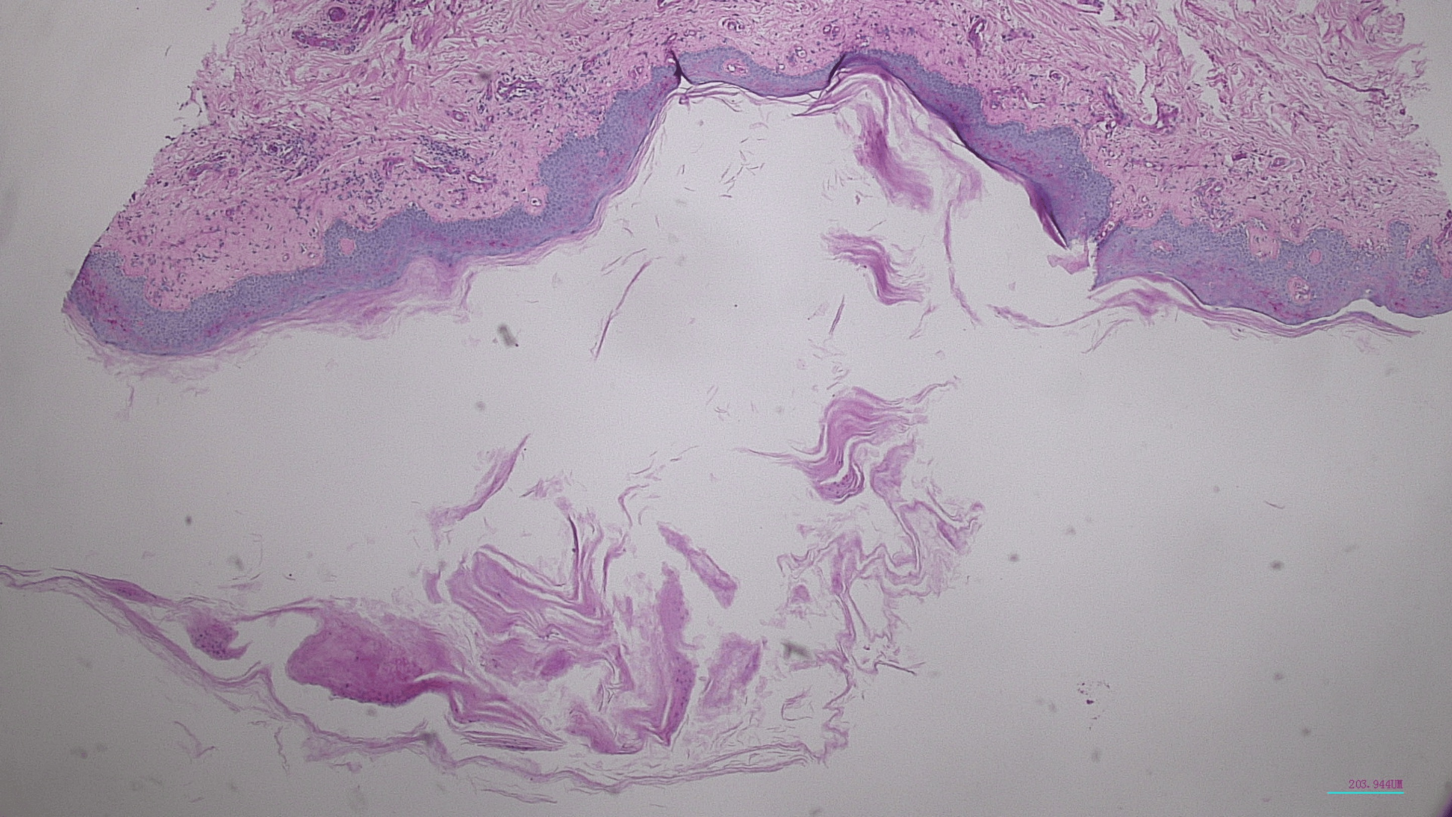
Histological image of a skin biopsy (hematoxylin and eosin stain, ×50) showing mild acanthosis, focal dry parakeratosis, and moderate dermal congestion, consistent with guttate psoriasis

**Figure 3:**
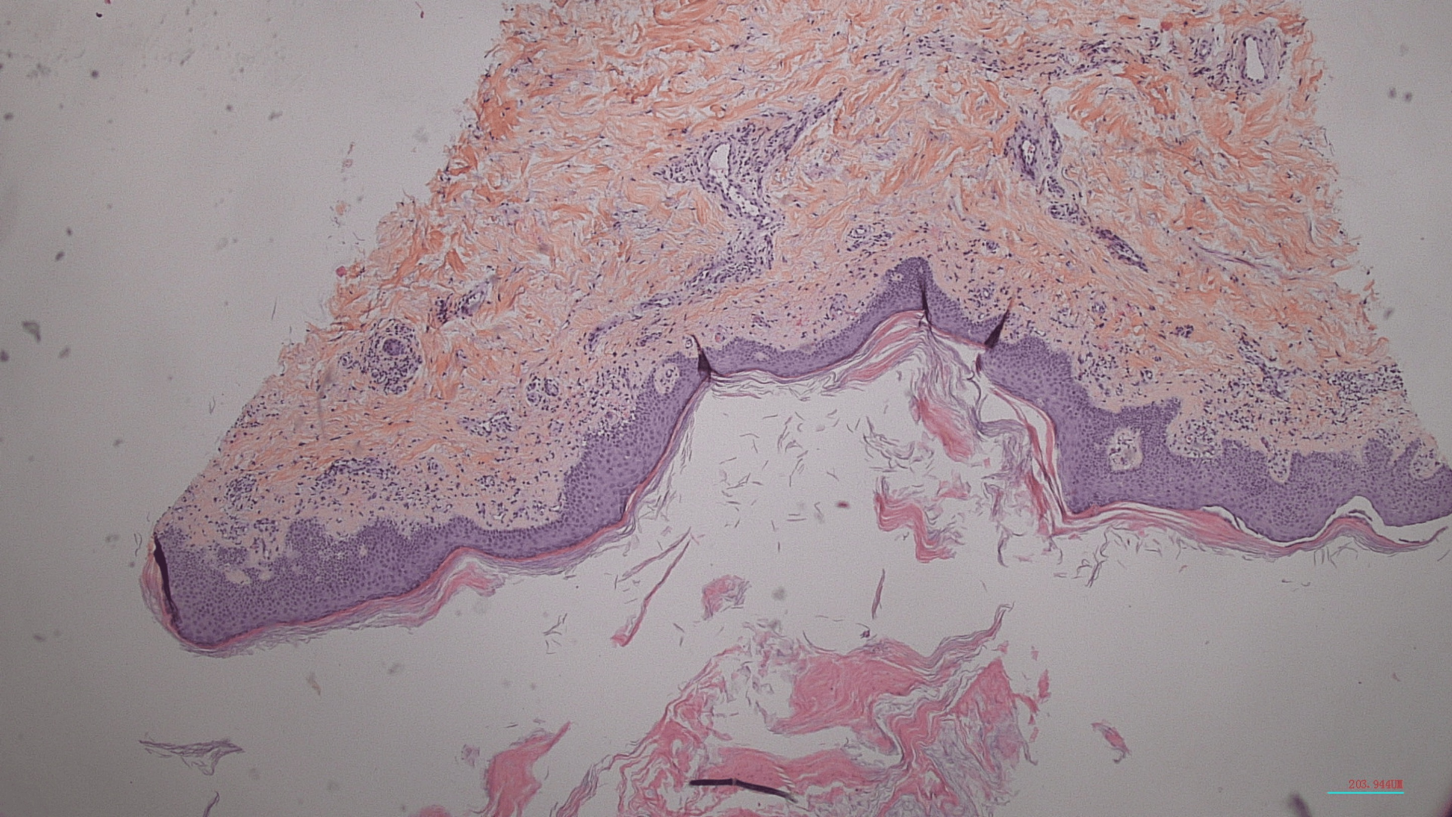
Periodic acid–Schiff (PAS) staining was negative on the skin biopsy (magnification ×50), showing no fungal elements, thereby ruling out dermatophytosis and supporting the diagnosis of psoriasis

Given the thin scaling observed, a skin biopsy of a lesion on the right leg was performed prior to any treatment in order to confirm the diagnosis. Histological analysis revealed mildly acanthotic epidermis with an irregular granular layer, focal dry parakeratosis containing rare polymorphonuclear cells, and mildly congested dermal papillae (Figure 2).​​​ Periodic acid-Schiff (PAS) staining revealed no fungal elements (Figure 3).

A topical regimen combining once-daily clobetasol propionate shampoo, once-daily calcipotriol/betamethasone dipropionate foam, and once-daily desonide cream led to a 50% reduction in lesions within one month. Durvalumab and bisoprolol treatments were continued without exacerbation of the lesions. Clinical follow-up at three months showed no worsening; the lesions remained stable.

## Discussion

Several cases of ICI-induced psoriasis have been reported [10,11], including a few with durvalumab. The median time to onset for durvalumab-induced psoriasis is 54.5 days (range: 7-690) [12]. This may represent either a flare of pre-existing psoriasis or a de novo eruption [5]. The proposed mechanism involves T-cell disinhibition - particularly Th1 and Th17 subsets - resulting in excessive production of pro-inflammatory cytokines, such as IL-17 and IL-23, which promote keratinocyte proliferation and inflammation [5].

Beta-blockers are also known to induce or exacerbate psoriasis, likely through inhibition of epidermal beta-adrenergic receptors, leading to reduced intracellular cyclic adenosine monophosphate (cAMP) and subsequent keratinocyte hyperproliferation [6]. Although bisoprolol is cardioselective, it has been associated with drug-induced psoriasis with variable latency, up to 12 months after initiation [13].

In our case, the short latency of 10 days following durvalumab initiation and the absence of lesions under bisoprolol alone suggest a primary causal role for durvalumab. Causality assessment using the Naranjo algorithm indicated a probable association between durvalumab and the onset of the psoriasiform eruption [14]. A synergistic effect with bisoprolol remains hypothetical. Continuation of durvalumab without modification is consistent with American Society of Clinical Oncology (ASCO)/European Society for Medical Oncology (ESMO) guidelines for moderate dermatologic irAEs, which recommend topical management without interruption of oncologic therapy [15,16].

Durvalumab was safely maintained under dermatologic supervision. The patient’s condition remained stable, with a 50% reduction in lesions and improved quality of life. This outcome reflects the importance of preserving essential therapeutic agents for patient well-being.

This case highlights the need to consider ICI-induced psoriasis in the differential diagnosis of new cutaneous eruptions during immunotherapy. It also underscores the importance of objective dermatologic evaluation using validated scoring systems (PASI, IGA, BSA), and the need for further research into the underlying mechanisms, incidence, risk factors, and optimal management strategies for ICI-related cutaneous adverse events.

## Conclusions

This case illustrates a de novo guttate psoriasis likely induced by durvalumab, with a possible contributory role of bisoprolol. The temporal relationship, absence of prior history, and favorable response to treatment support a probable causal association. Conservative management allowed immunotherapy to be safely maintained. Further research is warranted to explore potential interactions between immune checkpoint inhibitors and beta-blockers, such as bisoprolol, particularly regarding underlying immunopathological mechanisms, the incidence of cutaneous adverse events, individual susceptibility factors, and optimal management strategies.
